# Molecular Dynamics Study of Ion Transport in Polymer Electrolytes of All-Solid-State Li-Ion Batteries

**DOI:** 10.3390/mi12091012

**Published:** 2021-08-26

**Authors:** Takuya Mabuchi, Koki Nakajima, Takashi Tokumasu

**Affiliations:** 1 Frontier Research Institute for Interdisciplinary Sciences, Tohoku University, 2-1-1 Katahira Aoba-ku, Sendai 980-8577, Miyagi, Japan; 2 Institute of Fluid Science, Tohoku University, 2-1-1 Katahira Aoba-ku, Sendai 980-8577, Miyagi, Japan; koki.nakajima.q4@dc.tohoku.ac.jp (K.N.); tokumasu@ifs.tohoku.ac.jp (T.T.); 3 Graduate School of Engineering, Tohoku University, 2-1-1 Katahira Aoba-ku, Sendai 980-8577, Miyagi, Japan

**Keywords:** molecular dynamics, polymer electrolyte, lithium-ion battery, salt concentration, hopping mechanism

## Abstract

Atomistic analysis of the ion transport in polymer electrolytes for all-solid-state Li-ion batteries was performed using molecular dynamics simulations to investigate the relationship between Li-ion transport and polymer morphology. Polyethylene oxide (PEO) and poly(diethylene oxide-alt-oxymethylene), P(2EO-MO), were used as the electrolyte materials, and the effects of salt concentrations and polymer types on the ion transport properties were explored. The size and number of LiTFSI clusters were found to increase with increasing salt concentrations, leading to a decrease in ion diffusivity at high salt concentrations. The Li-ion transport mechanisms were further analyzed by calculating the inter/intra-hopping rate and distance at various ion concentrations in PEO and P(2EO-MO) polymers. While the balance between the rate and distance of inter-hopping was comparable for both PEO and P(2EO-MO), the intra-hopping rate and distance were found to be higher in PEO than in P(2EO-MO), leading to a higher diffusivity in PEO. The results of this study provide insights into the correlation between the nanoscopic structures of ion solvation and the dynamics of Li-ion transport in polymer electrolytes.

## 1. Introduction

In recent years, with the increase in energy demand and the seriousness of environmental problems, the demand for a new energy production technology that does not use fossil fuels has been increasing. Li-ion batteries are drawing attention as next-generation power sources because of their small size, light weight, high energy density, and relatively high durability [1]. In recent years, numerous studies have investigated solid electrolytes that do not leak and have a relatively high degree of freedom in design [2]. Various types of solid electrolyte materials, such as sulfide-based or oxide-based inorganic solid electrolytes and polymer-based electrolytes, have been widely used. Among them, polymer electrolytes are drawing attention because of their resistance to mechanical shocks, such as vibration, and their excellent mechanical properties [3]. One such polymer electrolyte is poly-ethylene oxide (PEO) [4]. Volel et al. [5] used pulsed-field-gradient NMR (PFG-NMR) measurements to show that LiTFSI salts in PEO exhibit superior ionic conductivity compared to LiBF_4_ and LiClO_4_ salts. Pozyczka et al. [6] showed that the transport number decreased with increasing salt concentration but started to increase above a certain concentration. Devaux et al. [7] evaluated the effect of polymer molecular weight on ionic conductivity and transport number in the PEO-LiTFSI system and reported small changes in these parameters when the molecular weight was of the order of 10 kg/mol. In addition, a study attempted to improve the performance of the system by inserting additives into it [8].

Theoretical approaches, such as numerical simulations [9,10,11,12] and molecular dynamics (MD) simulations [13,14,15], have been extensively used to study ion transport phenomena. As the ion transport phenomenon in PEO is largely due to the nanoscale structure of polymer electrolytes, several studies using molecular dynamics (MD) simulations have been conducted to explore this phenomenon. Brooks et al. [13] performed MD simulations of the PEO-LiTFSI system and observed that Li ions in the polymer electrolyte are coordinated to oxygen atoms, and proposed three types of transport mechanisms: intra-hopping, inter-hopping, and co-diffusion. In intra-hopping, Li ions coordinated to oxygen atoms move along the polymer chain, whereas in inter-hopping, Li ions move to sites of other polymer chains or distant sites of the same polymer chains. In co-diffusion, Li ions move with the polymer chain while being coordinated at the same site. Webb et al. [14,15] calculated and compared the ionic conductivity in various polymer electrolytes, including PEO, using MD simulations but did not find a polymer material superior to PEO. They also showed that, in the PEO-LiTFSI calculation system, the nanoscale-order time handled by MD simulation is effective in the sub-diffusion region where the diffusion coefficient of ions becomes small. However, as the qualitative tendency of the difference in ion transportability between different polymer species is consistent with that in the diffusion coefficient at macroscale-order times, it was shown that a qualitative discussion on the difference in ion transportability between different polymer species was valid even in MD simulations with relatively small time scales. In recent years, Zheng et al. [16] proposed poly(diethylene oxide-alt-oxymethylene), P(2EO-MO), a polymer material that may exhibit better performance than PEO. They measured the Li salt concentration dependence of ionic conductivity for this polymer by the AC impedance method and evaluated the salt concentration dependence of transport number by the steady-state current technique. They reported that although the polymer was inferior to PEO in terms of ionic conductivity, it was superior in terms of transport number. However, the detailed transport mechanisms responsible for the differences in ionic conductivity and transport number have not yet been clarified.

Although the types of macromolecules and ions are different in the present study, in our previous studies, we analyzed the ion transport phenomena in polymer electrolyte membranes and polymer thin films with thicknesses of several nanometers in polymer electrolyte fuel cells using MD simulations; further, we analyzed the correlation of the polymer structure with the vehicular ion diffusion [17], the structural ion diffusion [18,19,20,21], and the electroosmosis [22]. Based on these findings, the present study aimed to clarify the effect of nanoscale structural properties on the ion transport properties of the PEO-LiTFSI system and P(2EO-MO)-LiTFSI system. While the Li-polymer co-diffusion contributes to the vehicular diffusion of lithium ions over short time scales, inter/intra-hopping is more important for the overall diffusion process [13]. Therefore, the present study focuses on investigating the inter/intra-hopping mechanisms. Our simulations provide quantitative information on the salt clusters and their relation to the inter/intra-hopping behaviors and the diffusivity, which to our knowledge has not been fully defined previously. The remainder of the paper is organized as follows. Section 2 describes the details of the calculation method, including the molecular model and calculation conditions used in this study. Section 3 describes the results of the salt concentration dependence on the correlation between structural properties, such as coordination structure around Li ions and cluster analysis in PEO and P(2EO-MO) electrolytes, and transport properties, such as diffusion coefficient, hopping frequency, and distance. Section 4 summarizes the results obtained in this study.

## 2. Simulation Methods

### 2.1. Molecular Models

**Figure 1:**
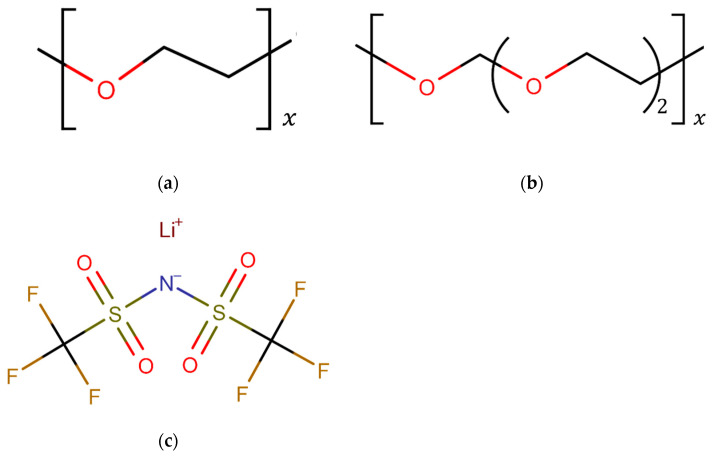
Structural formulas of (**a**) PEO, (**b**) (2EO-MO), and (**c**) the LiTFSI salt considered in the present simulations.

The structural formulas of PEO and P(2EO-MO) are shown in Figure 1a,b. Timachova et al. [23] conducted PFG-NMR experiments and reported that there was no significant change in the results of the diffusion coefficient and ionic conductivity when the molecular weight was approximately 10 kg/mol or more. Therefore, the number of monomers per polymer was set at 100 in PEO, in accordance with previous studies [13,24]. P(2EO-MO) was adjusted to have a similar number of oxygen atoms to PEO, with x = 33, providing a comparable number of lithium ions at the same salt concentration, so that the results of cluster analysis can be compared directly between PEO and P(2EO-MO) systems. For the intramolecular and intermolecular interactions of PEO and P(2EO-MO), the united-atom (UA) model [14,15], which treats CH_2_ and CH_3_ as one particle, was used. The CHARMM force field [25] was used for the bond parameter, and the TraPPE-UA force field [26] was used for the angle and torsion parameters. LiTFSI was used as the Li salt, and the non-polar all-atom model proposed by Wu et al. [27] was adopted. The structural formula of the Li salt is shown in Figure 1c.

### 2.2. Simulation Conditions

**Figure 2:**
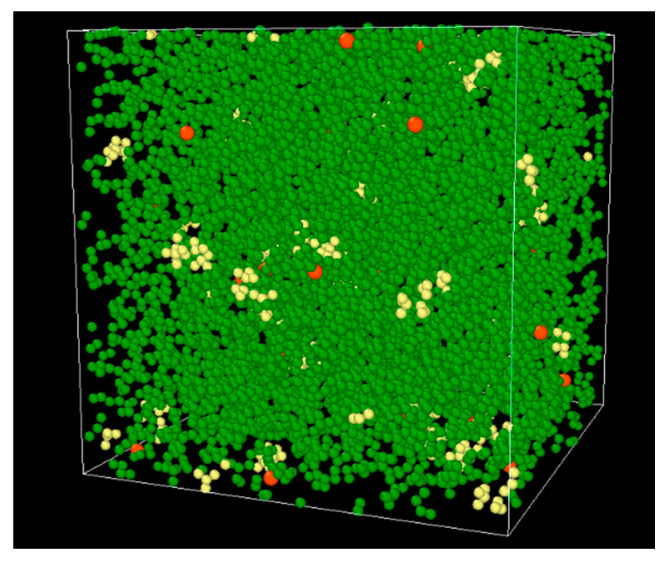
A snapshot of PEO system at a salt concentration of 0.01. PEO polymer is shown as green particles and Li and TFSI ions are shown as orange and yellow particles, respectively.

In this study, 30 chains were used for each system of PEO and P(2EO-MO), and the LiTFSI salt concentrations were set at *r* = ([Li])/([O]) = 0.01, 0.03, 0.08, and 0.12. Here, [Li] indicates the molar concentration of Li in the calculation system, and [O] indicates the molar concentration of the O atom of the polymer. The number of molecules of LiTFSI for each salt concentration is 30, 90, 240, and 360, respectively. These molecules were randomly arranged in the calculation area *x* × *y* × *z* = 360 × 360 × 360 Å^3^ to create an initial structure. Next, energy minimization was performed by the conjugate gradient algorithm [28], and then the most stable equilibrium state was achieved according to the annealing procedure proposed in a previous study [17]. Table 1 shows the density of the calculation systems after the annealing. Compared with the experimental PEO density value of 1.13 g/cm^3^, the system exhibited a relatively good agreement, confirming the validity of the model. For the density of P(2EO-MO), no experimental value was found; therefore, the validity of the calculation system was verified based on the structural information, which is described later. After annealing, the NPT simulations were carried out for 100 ps under the conditions of *P* = 1 atm and *T* = 400 K, after which the positions of all atoms were recorded at 0.1 ps intervals for 150 ns in the NVT ensemble under the condition of *T* = 400 K. Here, *N* is the number of molecules, *V* is the volume of the system, *P* is the pressure, and *T* is the temperature. The Nosé–Hoover method [29,30] was used for temperature control, and the Parrinello–Rahman method [31,32] was used for pressure control. The smooth particle mesh Ewald method [33] was used for calculating of the Coulomb force, and the cutoff distances of the LJ potential and the Coulomb potential were set at 15 Å. All calculations were performed using the LAMMPS package [34]. Figure 2 shows an example of a final-state snapshot of PEO system at *r* = 0.01.

## 3. Results and Discussion

### 3.1. Structural Characteristics around Li Ions

**Figure 3:**
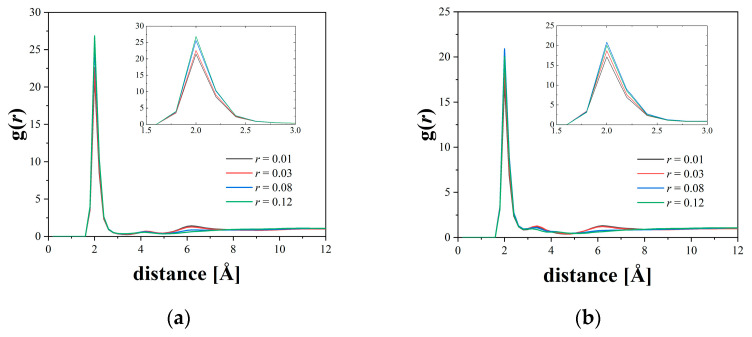
Radial distribution function between Li ions and polymer oxygen atoms at various salt concentrations for (**a**) PEO and (**b**) P(2EO-MO). The insets are magnified view of the first peak.

Figure 3 shows the results of the radial distribution function between the Li ions and the oxygen atoms of the polymer at different salt concentrations. For all the cases, Li ions were found to be coordinated and exist in the vicinity of 2 Å from the O atom of the polymer, which is in good agreement with the scattering experiment [35]. The small changes in the first peak height for the different salt concentrations corresponds to the small changes in the number of coordinated oxygens around a Li ion. This tendency was found to be in good agreement with the MD simulation results of Zheng et al. [16]. From these results, 3.25 Å, which corresponds to the first minimum, was used as the threshold value for determining whether the O atom is coordinated.

**Figure 4:**
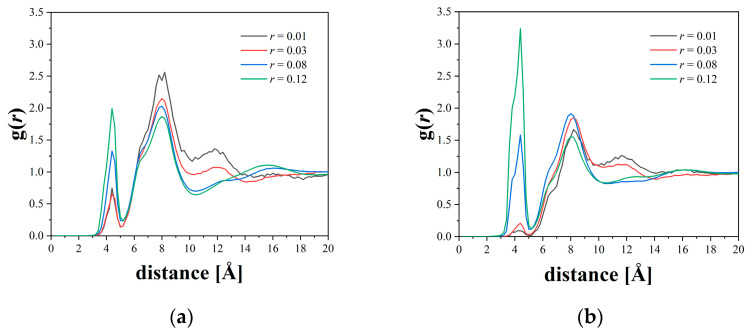
Radial distribution function between Li ions and N atoms of TFSI ions at various salt concentrations for (**a**) PEO and (**b**) P(2EO-MO).

Figure 4 shows the results of the radial distribution function between the N atoms of Li and TFSI ions at different salt concentrations. Because the first minimum was found to be at 5.0 Å for all the cases, this value was used as the threshold for determining whether LiTFSI is dissociated. The smaller peaks at low salt concentrations indicate that both Li and TFSI ions are well dissociated in both systems, which is consistent with the observation in the MD simulation by Zheng et al. [16]. The first peak was found to be higher at higher salt concentrations, indicating that the number of undissociated LiTFSI salts increased as the salt concentration increased. Particularly, a larger increase in the first peak height with increasing salt concentration was observed for the P(2EO-MO) system, indicating the Li and TFSI ions tend to form clusters at higher concentrations in the P(2EO-MO) system.

**Figure 5:**
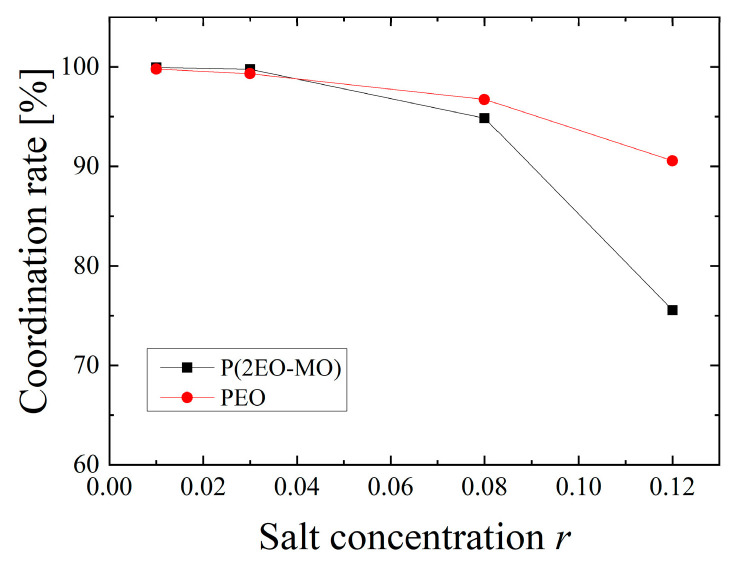
Oxygen coordination rate of Li ions.

From these results, it can be seen that the Li ion always has a strong bond with the TFSI ions and/or the oxygen atoms of the polymer. Because the oxygen coordination structure is important for the transport of Li ions by inter/intra-hopping, the results for the oxygen coordination rate under each condition were obtained, as shown in Figure 5. Here, the oxygen coordination rate β of Li ions is defined as the fraction of Li ions coordinated by oxygen atoms of five or more macromolecules among all Li ions. From the figure, it can be seen that the coordination rate decreases as the salt concentration increases. This trend is due to the LiTFSI salt formation at a high salt concentration and the decrease in the coordination number with the oxygen atoms.

### 3.2. Cluster Analysis

**Figure 6:**
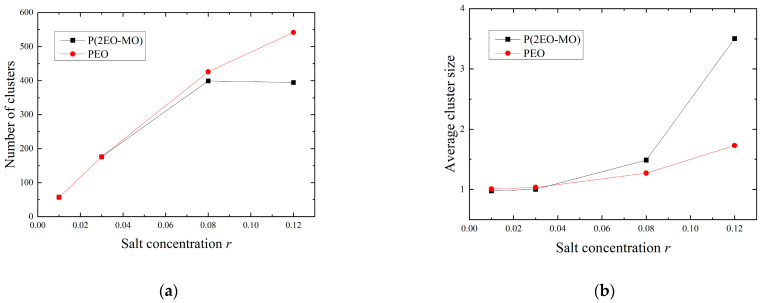
(**a**) Number of LiTFSI clusters and (**b**) average size of LiTFSI clusters.

The transport characteristics of Li ions are considered to be significantly affected by the number and size of clusters containing multiple Li ions and TFSI ions [6]. In this study, Li ions or aggregates of TFSI ions with a Li-TFSI distance of 5 Å or less are defined as LiTFSI clusters. The size of LiTFSI clusters is defined by the number of Li or TFSI ions contained within them; further, LiTFSI molecules that are not dissociated and exist as single LiTFSI molecules are also treated as LiTFSI clusters of size 2. The number and size of LiTFSI clusters are shown in Figure 6. In the high salt concentration range, PEO has more clusters; however, the average size is smaller. As the cluster size increases, the Li ions in the cluster are more susceptible to the influence of surrounding molecules. Therefore, the Li ions in P(2EO-MO), which forms relatively large clusters in high salt concentration regions, are expected to be more constrained by the influence of the surrounding TFSI salt than those in PEO, making the Li ions less mobile.

### 3.3. Transport Characteristics and Transport Number of Li Ions

**Figure 7:**
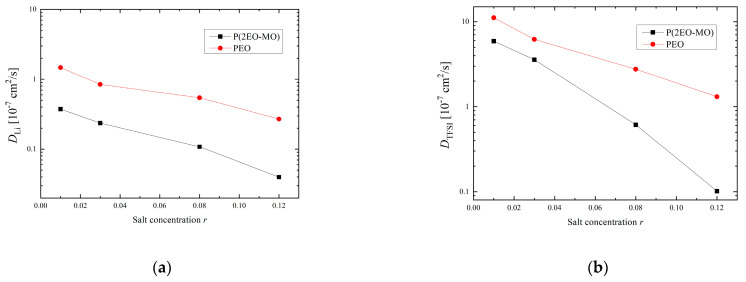
Salt concentration dependence of (**a**) Li ion and (**b**) TFSI ion diffusion coefficient.

**Figure 8:**
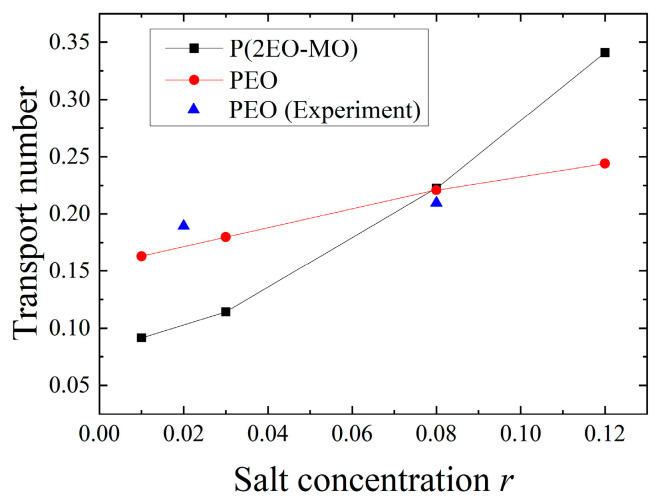
Salt concentration dependence of transport number. Experimental data were taken from Timachova et al. [23].

Figure 7a shows the results of the self-diffusion coefficient, calculated using the mean square displacement, for the transport characteristics of Li ions. To calculate the transport number, the self-diffusivity coefficient of TFSI ions was calculated in the same manner, and the corresponding results are shown in Figure 7b. It can be seen that the diffusion coefficient in PEO is high for all ions at any salt concentration and decreases with increasing salt concentration, regardless of the polymer species. These results are in good agreement with the experimental results reported by Zheng et al. [16], which showed that PEO has higher ionic conductivity than that of P(2EO-MO) at any salt concentration. In addition, as compared to the trends of PEO, the diffusion coefficient of both ions in P(2EO-MO) decreases significantly and the difference in diffusion coefficient owing to the difference in polymer species increases as the salt concentration increases. This result is consistent with the fact that P(2EO-MO) forms a relatively large LiTFSI cluster, particularly in the high-salt-concentration range; it is suggested that the LiTFSI clusters constrain the ions to each other and reduce diffusivity. Furthermore, based on these diffusion coefficients, the transport number was calculated using the following formula:

(1) $$t_{+}=\frac{D_{{\mathrm{Li}}^{+}}}{D_{{\mathrm{Li}}^{+}}+D_{{\mathrm{TFSI}}^{-}}}$$

where, $D_{{\mathrm{Li}}^{+}}$ indicates the diffusion coefficient of Li ions, and $D_{{\mathrm{TFSI}}^{-}}$ indicates the diffusion coefficient of TFSI ions. Figure 8 shows the salt concentration dependence of the transport number, $t_{+}$. The results are in good agreement with the experimental results reported by Timachova et al. [23], and the validity of this calculation was verified. The higher the salt concentration, the higher the transport number, regardless of the polymer species. However, the transport number of P(2EO-MO) increased relatively significantly, and the transport number of P(2EO-MO) was higher than that of PEO at high salt concentrations. This trend is attributed to the larger decrease in the diffusion coefficient of TFSI at a high salt concentration in P(2EO-MO) than that in PEO.

To clarify the transport mechanism in detail, the diffusion coefficient was decomposed using the oxygen coordination ratio β of Li ion, shown in Figure 5, as follows:

(2) $$D_{\mathrm{Li}}=D_{\mathrm{Li}\in O}\times \beta +D_{\mathrm{Li}\notin O}\times (1-\beta )$$

where, $D_{\mathrm{Li}\in O}$ and $D_{\mathrm{Li}\notin O}$ indicate the diffusion coefficient of Li ions coordinated, and not coordinated to the oxygen atoms, respectively. Furthermore, based on $D_{\mathrm{Li}\in O}$, among the Li ions coordinated to the oxygen atom, the ions that are dissociated from TFSI and those that are part of the LiTFSI cluster can be classified. However, β is relatively high (75% or more) under all conditions, as shown in Figure 5, and the effect of $D_{\mathrm{Li}\notin O}$ on $D_{\mathrm{Li}}$ is relatively small.

### 3.4. Hopping Analysis

**Figure 9:**
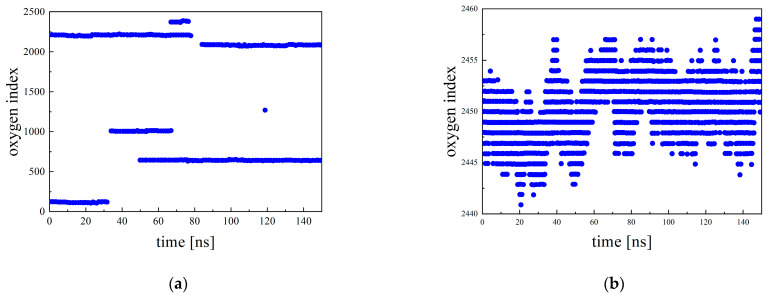
Examples of oxygen index as a function of time for (**a**) P(2EO-MO) at *r* = 0.01 and (**b**) PEO at *r* = 0.12.

For $D_{\mathrm{Li}\in O}$, the inter/intra-hopping number and distance are thought to have a large effect on the dissociated Li ions. For Li ions that are part of the cluster, the number and size of LiTFSI clusters may have an effect in addition to the inter/intra-hopping number and distance. For $D_{\mathrm{Li}\notin O}$, only the number and size of LITFSI clusters are considered to have an effect. In this study, we analyzed the effect of inter/intra-hopping on $D_{\mathrm{Li}\in O}$. The indices of oxygen atoms that are within 3.25 Å of the lithium cation were used to determine inter/intra-hopping. In particular, each oxygen atom of the polymers was labeled sequentially, starting at one end of a polymer chain and continuing to the end of that chain before proceeding to the next; the oxygen atoms were consecutively labeled from 1 to 3000 for PEO and from 1 to 2970 for P(2EO-MO). This index was outputted every 100 ps, and whether Li ions had caused inter/intra-hopping was determined based on the change in the oxygen index. Because the typical Li ion is coordinated with around five oxygens, changes in the oxygen index were smaller than five when a Li ion moves to the next oxygen site along the chain where new coordinated oxygens include partly the previous ones. Therefore, we used this threshold to define the inter/intra-hopping in this study. In other words, when the change was between one and five, it was judged that intra-hopping occured, and when the change was five or more, it was judged that inter-hopping occured. Examples of the oxygen index when inter- and intra-hopping occur are shown in Figure 9. Figure 9a shows the results for P(2EO-MO) at *r* = 0.01, while Figure 9b shows the results for PEO at *r* = 0.12, respectively. In Figure 9a, the oxygen index changes significantly around 35 ns, indicating that inter-hopping occurred. In Figure 9b, it can be seen that relatively small changes in the oxygen index occurred intermittently, indicating that intra-hopping occurred frequently.

**Figure 10:**
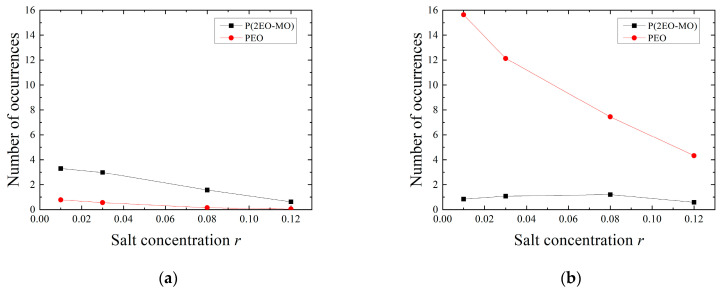

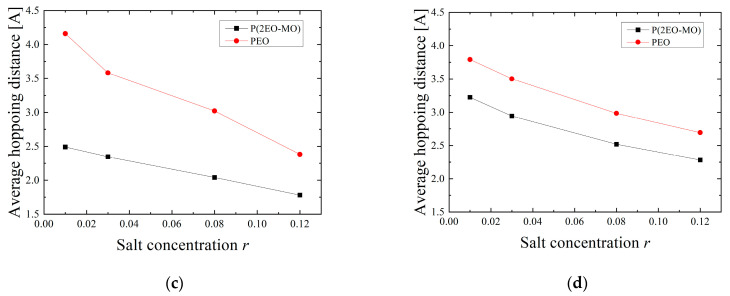
Number of occurrences of (**a**) inter-hopping and (**b**) intra-hopping. Average distance of (**c**) inter-hopping and (**d**) intra-hopping.

Next, the number of occurrences of inter/intra-hopping and the average hopping distance were calculated. The number of occurrences of hopping per Li ion was calculated by dividing the total number of occurrences by the number of Li ions coordinated to the O atom. The average hopping distance was calculated by dividing the total hopping distance by the total number of occurrences. The results of the salt concentration dependence of the number of hopping occurrences and the distance in each polymer are shown in Figure 10. It can be seen that the number of occurrences decreases, and the distance becomes shorter as the salt concentration increases, regardless of the type of hopping and the type of polymer. The fraction of Li ions among the Li ions coordinated to the O atoms that also belong to LiTFSI clusters increases as the salt concentration increases; therefore, the Li ions are likely to be constrained to the cluster at high salt concentrations. Thus, the number of occurrences of hopping is considered to have decreased. Furthermore, it is thought that the hopping distance decreased because the average distance between Li ions decreased at high salt concentration; as a result, Li ions were more susceptible to inhibition by other Li ions when hopping. This phenomenon led to a decrease in the diffusion coefficient $D_{\mathrm{Li}\in O}$ of the Li ions coordinated to the oxygen atoms and, therefore, a decrease in the diffusion coefficient of the entire Li ion. Regarding the effect of polymer species on the diffusion coefficient, it can be confirmed that PEO has a relatively smaller number of occurrences of inter-hopping compared to P(2EO-MO), but the hopping distance is larger. Therefore, there is no significant difference between polymer species in terms of the effect of inter-hopping on diffusivity. In the case of intra-hopping, PEO has a larger number of occurrences and greater distance compared to those in P(2EO-MO), leading to the larger diffusion coefficient of PEO.

## 4. Conclusions

We analyzed the ion transport inside an all-solid-state lithium-ion battery polymer electrolyte membrane using MD simulations. We evaluated the salt concentration dependence of ion transport and structural properties in the electrolyte. Furthermore, we examined the difference in transport mechanism in each electrolyte membrane using PEO, a representative polymer electrolyte material, and P(2EO-MO), which was proposed as a new material. From the results of oxygen coordination structure around Li ions and LiTFSI cluster analysis, it was shown that clusters comprising multiple LiTFSI salts are formed as the salt concentration increases. It was suggested that P(2EO-MO) limits the movement of Li ions by forming relatively large clusters in the high-salt-concentration region as compared to PEO. From the diffusion coefficient and hopping analysis, it was confirmed that the diffusion coefficients of both the Li ion and the TFSI ion were superior to that of PEO, which was in good agreement with the experimental results. Furthermore, the transport number also showed good agreement with the experimental results, and the validity of this calculation was verified. Moreover, the number of occurrences of hopping decreased and the distance became shorter as the salt concentration increased, regardless of the type of hopping and the type of polymer. In particular, regarding intra-hopping, PEO had a larger number of hopping events and greater hopping distance, leading to the larger diffusion coefficient of PEO.

## Figures and Tables

**Table 1:** Density after annealing [g/cm^3^].

Salt Concentration, *r*	0.01	0.03	0.08	0.12
PEO	1.09	1.16	1.28	1.37
P(2EO-MO)	1.16	1.22	1.35	1.44
